# A big white dot after CPR

**DOI:** 10.1186/s12245-021-00376-3

**Published:** 2021-09-10

**Authors:** Sushma Kola, Alexander D. Ginsburg, Laura Harper, Laura E. Walker, Sherri Braksick, Eelco F. M. Wijdicks

**Affiliations:** 1 Department of Neurology, Mayo Clinic, 200 First Street, SW, Rochester, MN 55902 USA; 2 Department of Emergency Medicine, Mayo Clinic, 200 First Street, SW, Rochester, MN 55902 USA; 3 Department of Radiology, Mayo Clinic, 200 First Street, SW, Rochester, MN 55902 USA

**Keywords:** Neuroradiology, MRI, Interventional, Stroke, Cerebrovascular disease

## Abstract

**Introduction:**

Patients may remain comatose after the resumption of spontaneous circulation with cardiopulmonary resuscitation. A primary neurologic event may precede a cardiac standstill.

**Case report:**

We present a 33-year-old patient with successful resuscitation for pulseless electrical activity and a “normal computed tomography (CT) scan.” Further scrutiny showed a hyperdense basilar artery sign (‘big white dot’) that led to a CT angiogram confirming an embolus to the proximal basilar artery. His examination showed fixed and dilated midsize (mesencephalic) pupils and extensor posturing. Endovascular retrieval of the clot was successful, but there was a devastating ischemic injury to the brainstem.

**Conclusion:**

This case reminds us to consider neurologic causes of cardiac arrest.

## Key points

1. A non-shockable rhythm in the setting of a sudden cardiac arrest should raise alarms for a primary non-cardiac ethology, and the absence of brainstem reflexes increases the likelihood of an intracranial process.
2. Ischemic stroke and intracranial hemorrhage are known, but rare, causes of out-of-hospital cardiac arrest leading to the development of non-shockable rhythms such as asystole or pulseless electrical activity.
3. Basilar artery occlusion suggested on non-contrast CT scan should be confirmed by a CT angiogram of the head and neck, followed by emergent mechanical thrombectomy.
4. In patients presenting with posterior circulation stroke-like symptoms, a hyperdense basilar artery sign on a non-contrast head CT is a strong predictor of basilar artery thrombosis.

## Introduction

Acute embolus to the basilar artery is one of the most devastating types of stroke. It may start insidiously and with vacillating signs of brainstem dysfunction or acutely result in a coma with extensor posturing. We report a patient presenting in pulseless electrical activity with the prior onset of acute emesis, vertigo, and diaphoresis.

## Case presentation

A 33-year-old male with a 20 pack-year smoking history and untreated hypertension was brought to the emergency department after a witnessed cardiac arrest. Limited history was initially available, but paramedics (EMS) reported that he had been vomiting earlier in the day and later collapsed. Bystander cardiopulmonary resuscitation was initiated, and EMS found the initial rhythm to be pulseless electrical activity (PEA) arrest. EMS continued chest compressions and provided a single dose of epinephrine via intraosseous access with the return of spontaneous circulation. He remained unresponsive, was intubated without sedating medication, and was transported to the hospital.

Upon arrival at the emergency department (ED), the patient had a blood pressure of 249/149, a heart rate of 114/min, and was not on a vasoactive agent. Oxygen saturations were appropriate and end-tidal CO2 was elevated. His pupils were questionably reactive, and he had no purposeful movements. He received a paralytic to facilitate effective ventilation.

Given his presenting symptoms, common causes of PEA arrest were considered, but initial evaluation did not identify a clear cause of the patient’s arrest. Point of care and bedside testing revealed normal potassium and hematocrit, as well as a mixed metabolic and respiratory acidosis with pH 6.99, CO2 94, and lactate 9.06. Subsequent laboratory tests included a normal serum troponin, serum toxicology, and COVID-19 PCR. Urine toxicology was presumptive positive for tetrahydrocannabinol.

**Fig. 1:**
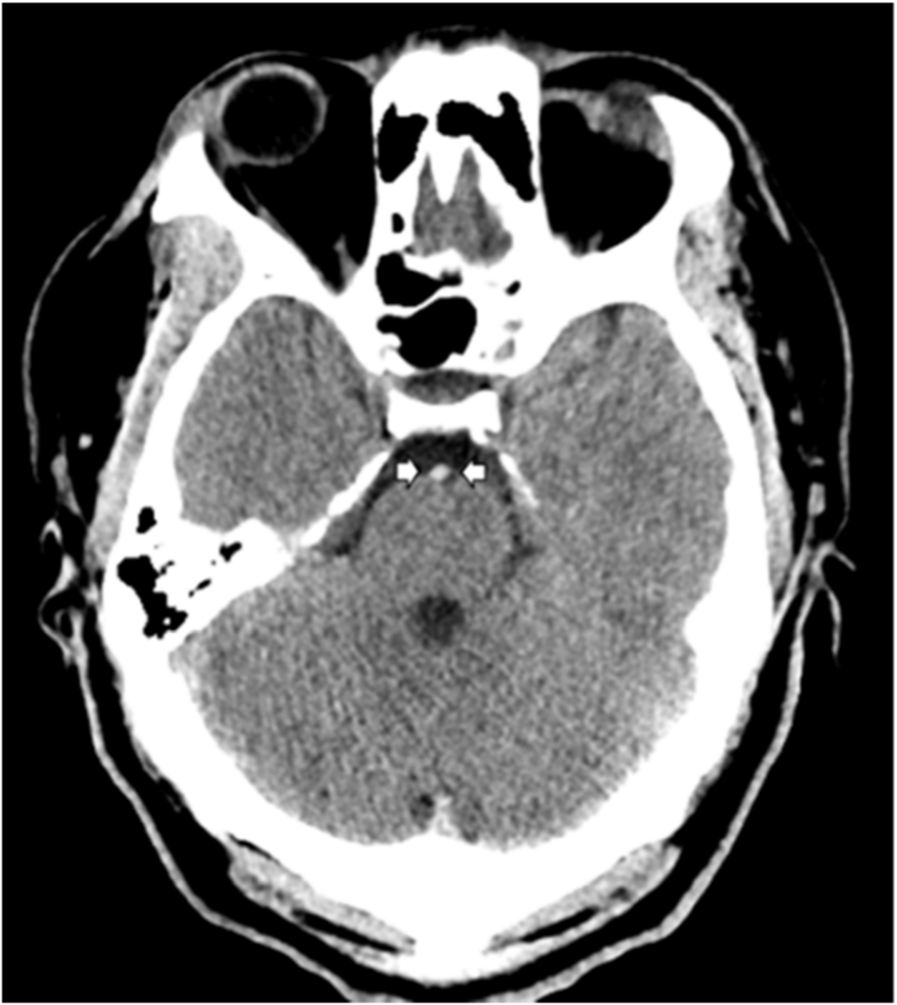
Non-contrast CT scan with hyperdense basilar artery sign (arrows). No hypodensities were identified in the brainstem, cerebellum, or cerebral hemispheres

A non-contrast CT of the head was initially thought to be normal, but upon further scrutiny, there was concern for a hyperdense basilar artery sign (Fig. 1).

**Fig. 2:**
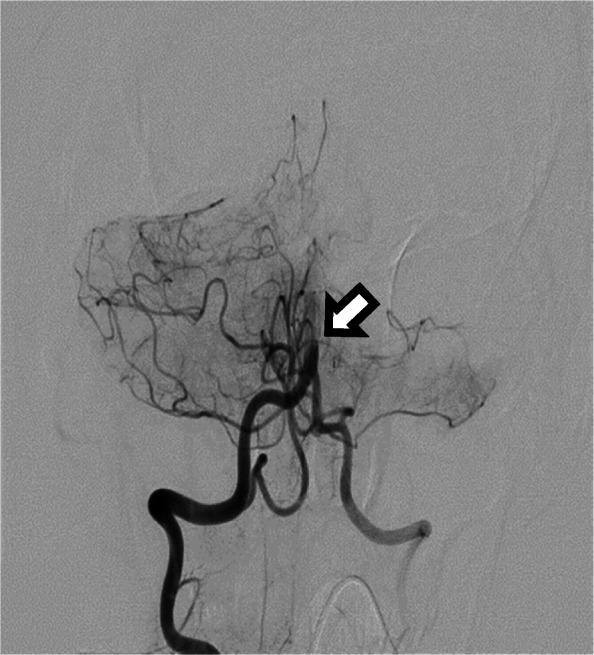
Cerebral angiogram showing proximal basilar artery occlusion (arrow)

CT angiogram of the head and neck demonstrated a complete basilar artery occlusion (BAO) at the vertebrobasilar junction (Fig. 2).

Additional history was obtained from the patient's partner after initial stabilization. The patient was noted to have a 20 pack-year smoking history and untreated hypertension. He was last known to be normal seven hours before arrival, before experiencing acute emesis, vertigo, and diaphoresis. Symptoms progressed to include dysphagia, ataxia, and left hemiparesis. Three hours later, he developed a right facial droop and dysarthria, and then he fell. EMS was ultimately called, and before their arrival, the patient collapsed and became unresponsive.

**Fig. 3:**
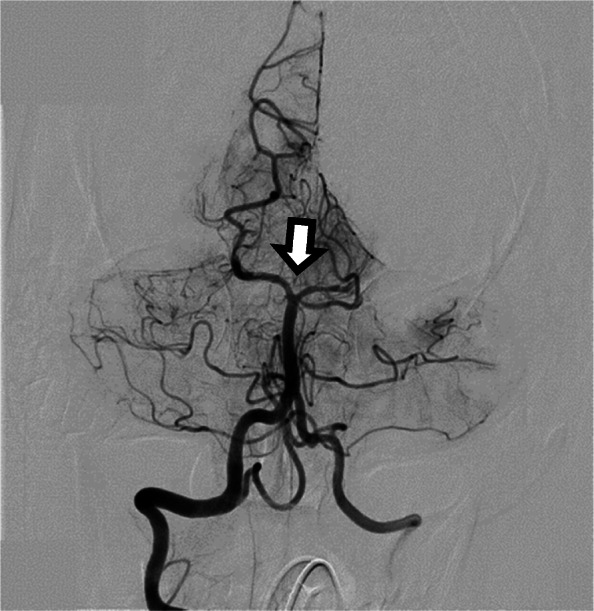
Recanalization of proximal basilar artery occlusion (arrow) with persistent bilateral PCA occlusions.

Due to the time of onset, the patient was not considered a candidate for intravenous thrombolysis. He emergently underwent mechanical embolectomy with successful recanalization of the basilar artery (Fig. 3).

**Fig. 4:**
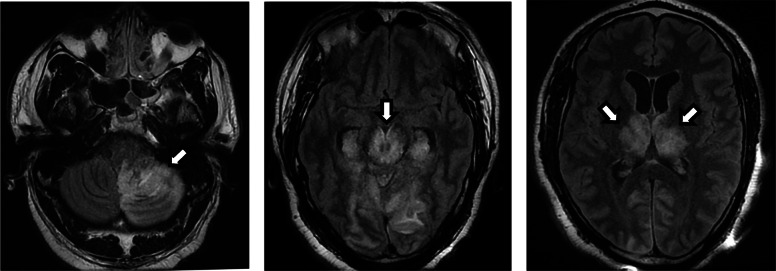
MRI brain without contrast shows severe ischemic injury of cerebellum, brainstem, and thalami (arrows).

Unfortunately, there was persistent occlusion of the distal bilateral posterior cerebral arteries (PCA). Multiple attempts at recanalization were unsuccessful, and the patient was taken to the intensive care unit for further management. Magnetic resonance imaging (MRI) of the brain without contrast demonstrated extensive posterior circulation infarcts, mass effect with effacement of the basilar cisterns, and obstructive hydrocephalus (Fig. 4).

Due to worsening neurologic examination, loss of upper brainstem reflexes, and poor prognosis, the family elected to pursue comfort measures.

## Discussion

The evaluation of PEA arrest typically focuses on conditions that can be treated and reversed rapidly to obtain the return of spontaneous circulation. Respiratory arrest often is followed by PEA, and in this case, it was a consequence of an acute neurologic event. Our case presentation is notable for (an unfortunately common) lack of detailed history and key neuroradiology findings on CT scan which must be recognized immediately. In patients presenting with posterior circulation stroke-like symptoms, a hyperdense basilar artery sign on the non-contrast head CT is a strong predictor of basilar artery thrombosis. Often, the diagnosis of a BAO comes before a patient history is obtained, as in our case. This serves as a reminder to consider neurologic causes of PEA arrest.

Studies looking at all underlying causes of PEA have revealed that up to 6.9% are due to intracranial hemorrhage, with subarachnoid hemorrhage being the most common. In patients with a cerebrovascular cause of cardiac arrest, prodromes were mostly neurologic and the mortality rate was 100% with brain death as the leading cause [1]

There is an association between all causes of cardiac arrest and ischemic stroke, but this occurs in fewer than 4% of cases [2]. A direct correlation between PEA arrest and BAO has not been reported in detail before. However, the deficits due to brainstem dysfunction may increase the risk of PEA arrest. Specifically, oropharyngeal dysfunction may increase the risk of obstruction, aspiration, and subsequent hypoxia progressing to PEA. Impairment of the respiratory drive may also contribute to respiratory failure.

## Conclusion

Diagnosis, in this case, was confounded by the patient’s unresponsiveness and loss of exam secondary to the administration of paralytics to facilitate ventilation. The subsequent history provided by the significant other included signs and symptoms concerning BAO. Vertigo, vomiting, dysphagia, dysarthria, ataxia, alternating hemiparesis, and gait unsteadiness are all suggestive of vertebrobasilar artery occlusion. Expeditious non-contrast CT head and CT head and neck angiogram could hasten the diagnosis and transport to a neuro-intervention suite to provide maximal chances of neurological recovery. If there are no perspicuous localizing neurological signs (as in this case), the discovery of a neurologic cause of cardiac arrest may be somewhere between heuristic reasoning and pure luck.

## Data Availability

Not applicable (images available)
